# The Rationality of Four Metrics of Network Robustness: A Viewpoint of Robust Growth of Generalized Meshes

**DOI:** 10.1371/journal.pone.0161077

**Published:** 2016-08-12

**Authors:** Xiaofan Yang, Yuanrui Zhu, Jing Hong, Lu-Xing Yang, Yingbo Wu, Yuan Yan Tang

**Affiliations:** 1 School of Software Engineering, Chongqing University, Chongqing, 400044, P.R. China; 2 Department of Computer Science, Georgia Institute of Technology, Atlanta, GA 30309, United States of America; 3 Faculty of Electrical Engineering, Mathematics and Computer Science, Delft University of Technology, 2600 GA Delft, The Netherlands; 4 Department of Computer and Information Science, University of Macau, Avenida da Universidade, Taipa, Macau, P.R. China; Universidad Rey Juan Carlos, SPAIN

## Abstract

There are quite a number of different metrics of network robustness. This paper addresses the rationality of four metrics of network robustness (the algebraic connectivity, the effective resistance, the average edge betweenness, and the efficiency) by investigating the robust growth of generalized meshes (GMs). First, a heuristic growth algorithm (the Proximity-Growth algorithm) is proposed. The resulting proximity-optimal GMs are intuitively robust and hence are adopted as the benchmark. Then, a generalized mesh (GM) is grown up by stepwise optimizing a given measure of network robustness. The following findings are presented: (1) The algebraic connectivity-optimal GMs deviate quickly from the proximity-optimal GMs, yielding a number of less robust GMs. This hints that the rationality of the algebraic connectivity as a measure of network robustness is still in doubt. (2) The effective resistace-optimal GMs and the average edge betweenness-optimal GMs are in line with the proximity-optimal GMs. This partly justifies the two quantities as metrics of network robustness. (3) The efficiency-optimal GMs deviate gradually from the proximity-optimal GMs, yielding some less robust GMs. This suggests the limited utility of the efficiency as a measure of network robustness.

## 1 Introduction

Nowadays, we live in a highly networked world, where numerous critical facilities are connected together by various networks. For the critical facilities to work properly, the underlying networks must be robust, that is, the performance of the surviving network degrades gradually with the increase of failing nodes/edges in such a network. To measure the robustness of a network, a number of metrics, such as the connectivity [1], the algebraic connectivity [2–7], the effective resistance [8–10], the average edge betweenness [9, 10], and the efficiency [10], have been proposed. However, the rationality of these metrics of network robustness is still in question.

In the process of infrastructural construction, it is often required that the robustness of an existing network be enhanced by adding an additional set of edges. Mosk-Aoyama [11] proved that the problem of maximizing the algebraic connectivity of an edge-growing network is NP-hard. Ghosh and Boyd [12] proposed a heuristic algorithm for solving the problem. Wang and Van Mieghem [13] designed and compared two heuristic algorithms for stepwise maximizing the algebraic connectivity of an edge-growing network. Abbas and Egerstedt [14] solved the problem of stepwise minimizing the effective resistance of an edge-growing network starting from an empty network. Wang et al. [15] proposed and compared four heuristic algorithms for stepwise minimizing the effective resistance of an edge-growing network starting from any given network. In the construction of infrastructures, it is also required that new nodes be linked to an existing network so as to form a most robust network. To our knowledge, however, problems of this sort have not yet being addressed.

A generalized mesh (GM) is a network that has a finite subset of integral points in the plane as the node set, where two nodes are adjacent if and only if they are one unit of Euclidean distance apart. GMs have widespread applications in areas such as parallel computing [16, 17], fault-tolerant communication [18–20], optical communication [21], city planning [22], percolation theory [23], and network epidemics [24–29].

This paper addresses the rationality of the four metrics of network robustness (the algebraic connectivity, the effective resistance, the average edge betweenness, and the efficiency) by investigating the robust growth of generalized meshes (GMs). First, a heuristic growth algorithm (the Proximity-Growth algorithm) is proposed. The resulting proximity-optimal GMs are intuitively robust and hence are adopted as the benchmark. Then, a generalized mesh (GM) is grown up by stepwise optimizing a given measure of network robustness. The following findings are presented: (1) The algebraic connectivity-optimal GMs deviate quickly from the proximity-optimal GMs, yielding a number of less robust GMs. This hints that the rationality of the algebraic connectivity as a measure of network robustness is still in doubt. (2) The effective resistace-optimal GMs and the average edge betweenness-optimal GMs are in line with the proximity-optimal GMs. This partly justifies the two quantities as metrics of network robustness. (3) The efficiency-optimal GMs deviate gradually from the proximity-optimal GMs, yielding some less robust GMs. This suggests the limited utility of the efficiency as a measure of network robustness.

The subsequent materials are organized in this fashion. Section 2 provides the preliminary knowledge. Section 3 describes a heuristic growth algorithm of networks. Sections 4–7 address the rationality of four different metrics of network robustness by examining the robustness of the corresponding node-growing networks, respectively. Finally, Section 8 summarizes this work.

## 2 Preliminary knowledge

### 2.1 Graph theory

For fundamental knowledge on graph theory, see Ref. [1]. Given a node *u* of graph *G*, let *d*_*G*_(*u*) denote the degree of *u* in *G*, and let $d_{G}^{\left(k\right)}\left(u\right)$ denote the number of nodes of *G* that are each distance *k* apart from *u* in *G*. Clearly, $d_{G}^{\left(1\right)}\left(u\right)=d_{G}\left(u\right)$.

**Definition 1**. *Let G = (V,E) be a graph*, *S* ⊆ *V*.

1. *S is referred to as a* separating set *of G if V* − *S is disconnected*.
2. *The connectivity of G, denoted* *κ*(*G*), *is defined as follows*.
  *κ*(*G*) = *min*{|*S*|: *S is a separating set of G*} *if G is not complete*;
  *κ*(*G*) = |*V*| − 1 *if G is complete*.

The connectivity is an early proposed measure of network robustness: a network with larger connectivity is intuitively more robust than a network with smaller connectivity. As one network may be intuitively more robust than another network with equal connectivity, it is necessary to introduce additional metrics of network robustness so as to fully capture the robustness of a network.

### 2.2 Mesh, infinite mesh and generalized mesh

**Fig 1:**
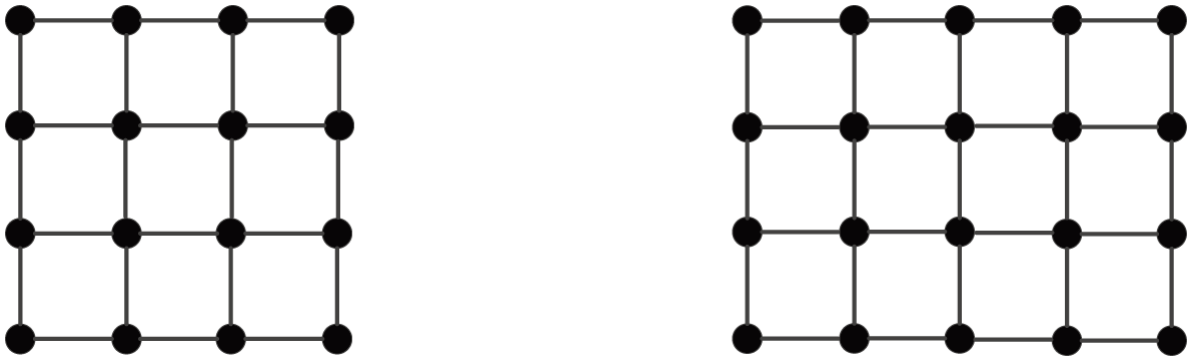
Two small-sized unlabeled meshes.

Let $Z_{n}=\left\{0,1,2,...,n-1\right\}$, Z={0,±1,±2,...}. An *m* × *n* mesh, denoted **M**_*m*×*n*_, is a graph with $Z_{m}\times Z_{n}$ as the node set, where two nodes, (*i*_1_,*j*_1_) and (*i*_2_,*j*_2_), are adjacent if and only if either (a) *i*_1_ = *i*_2_ and *j*_1_ = *j*_2_ ± 1, or (b) *j*_1_ = *j*_2_ and *i*_1_ = *i*_2_ ± 1. **M**_*n*×*n*_ is abbreviated as **M**_*n*_. Fig 1 depicts two small-sized unlabeled meshes.

**Fig 2:**
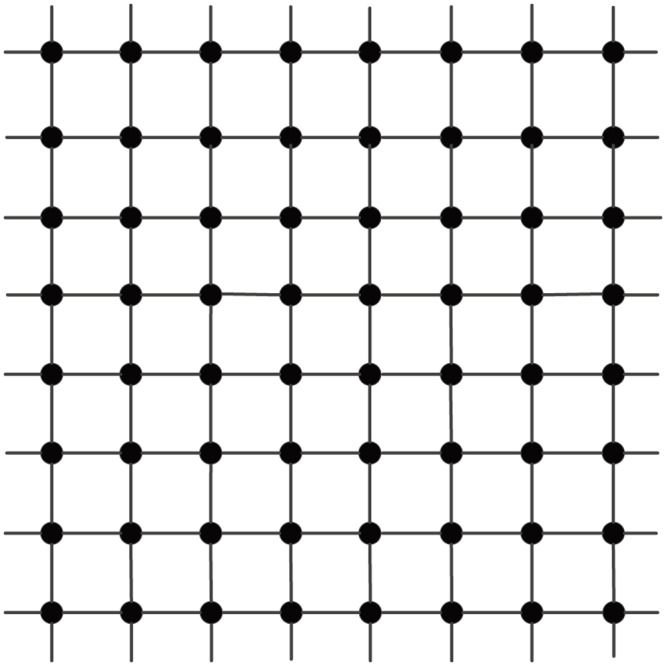
An infinite mesh.

An *infinite* *mesh*, denoted **M**_∞_, is an infinite graph with Z×Z as the node set, where two nodes, (*i*_1_,*j*_1_) and (*i*_2_,*j*_2_), are adjacent if and only if either (a) *i*_1_ = *i*_2_ and *j*_1_ = *j*_2_ ± 1, or (b) *j*_1_ = *j*_2_ and *i*_1_ = *i*_2_ ± 1. Fig 2 depicts an infinite mesh.

**Fig 3:**
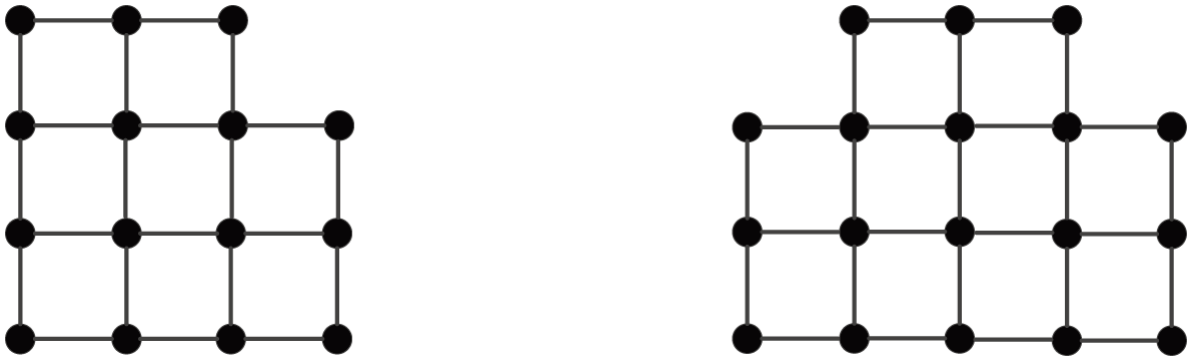
Two generalized meshes.

A *generalized* *mesh* (GM, for short) is a graph with a finite subset of $Z^{2}$ as the node set, where two nodes, (*i*_1_,*j*_1_) and (*i*_2_,*j*_2_), are adjacent if and only if either (a) *i*_1_ = *i*_2_ and *j*_1_ = *j*_2_ ± 1, or (b) *j*_1_ = *j*_2_ and *i*_1_ = *i*_2_ ± 1. Clearly, meshes are special GMs. Fig 3 depicts two GMs.

### 2.3 The robust growth of GMs

An initially small GM can grow up by stepwise adding new nodes. In real-world applications, it is often desired that the addition of a new node to a GM leads to a new GM with the best robustness. However, what the best robustness means is not clear, because there are quite a number of different metrics of network robustness. Indeed, given any metric of network robustness, *λ*, a GM can be grown up by adding a new node so that the resulting GM achieves the optimal *λ* value. Now, let us introduce some relevant notations and terminologies as follows.

**Definition 2**. *Given a metric of network robustness*, *λ*. *Consider a growth process of* **M**_2_ *by stepwise adding new nodes so that each resulting GM achieves the optimal λ value*.

1. *The process is referred to as a λ*-*optimal growth*.
2. *A sequence of GMs produced in this growth process is referred to as a λ*-*optimal sequence*.
3. *Each GM in a λ*-*optimal sequence is referred to as a λ*-*optimal GM. Let* **G** **M**_*λ*_(*N*) *denote a λ*-*optimal GM with N nodes*.

Sections 4–7 of this paper will examine the *λ*-optimal sequence, where *λ* is algebraic connectivity, effective resistance, average edge betweenness, and efficiency, respectively.

## 3 Heuristic growth of GMs

Given a generalized mesh *G*, define a sequence of subsets of $Z^{2}-V\left(G\right)$, *D*_1_, *D*_2_, …, recursively as follows.

1. $D_{1}=\{u\in Z^{2}-V\left(G\right):$ *d*_*G*+*u*_(*u*) attains the maximum; v ≇ w for any *v*,*w* ∈ *D*_1_, *v* ≠ *w*}.
2. For *k* ≥ 2, *D*_*k*_ = {*u* ∈ *D*_*k*−1_: $d_{G+u}^{\left(k\right)}\left(u\right)$ attains the maximum; v ≇ w for any *v*,*w* ∈ *D*_*k*_, *v* ≠ *w*}.

Clearly, *D*_1_ ⊇ *D*_2_ ⊇ ⋯, and $D_{\left|V(G)\right|}=⌀$.

Below let us describe a heuristic algorithm for the robust growth of GMs.

**Algorithm**: Proximity-Growth

**Input**: a generalized mesh *G*.

**Output**: a generalized mesh *G* + *u*, $u\in Z^{2}-V\left(G\right)$.

begin

 *k*: = 1;

 while *D_k_* ≠ ⌀,

  if |*D*_*k*_| = 1, let *D*_*k*_ = {*u*}, return(*u*);

  else *k*++;

 end while;

 arbitrarily choose *u* ∈ *D*_*k*−1_, return(*u*);

end

Intuitively, this algorithm grows a GM in a most robust way, because the newly added node is best connected to the GM. Clearly, the Proximity-Growth algorithm applies not only to GMs but to any other class of networks.

For our purpose, let us introduce the following notations and terminologies.

**Definition 3**. *Consider a growth process of* **M**_2_ *by repeatedly running the Proximity-Growth algorithm*.

1. *This process is referred to as a proximity growth*.
2. *A sequence of GMs produced in a proximity growth is referred to as a PR*-*sequence*.
3. *Each GM in a PR-sequence is referred to as a PR-GM. Let* **G** **M**_*PR*_(*N*) *denote a PR-GM with N nodes*.

Theoretical analysis reveals that, up to isomorphism, the proximity growth of GMs is as follows.

1. If the current GM is **M**_*n*_ and *n* is even, then grow **M**_*n*_ to **M**_*n*+1_ in the following node-adding order:
  $$\begin{array}{l} \left(n,\frac{n-2}{2})\to (n,\frac{n}{2})\to (n,\frac{n-4}{2})\to (n,\frac{n+2}{2})\to \ldots (n,0)\to (n,n-1\right)\\ (\frac{n}{2},n)\to (\frac{n-2}{2},n)\to (\frac{n+2}{2},n)\to (\frac{n-4}{2},n)\to (\frac{n+4}{2},n).\\ \to \ldots \to (0,n)\to (n,n). \end{array}$$
2. If the current GM is **M**_*n*_ and *n* is odd, then grow **M**_*n*_ to **M**_*n*+1_ in the following node-adding order:
  $$\begin{array}{l} \left(n,\frac{n-1}{2})\to (n,\frac{n-3}{2})\to (n,\frac{n+1}{2})\to (n,\frac{n-5}{2})\to (n,\frac{n+3}{2}\right)\\ \to \ldots \to (n,0)\to (n,n-1).\\ (\frac{n-1}{2},n)\to (\frac{n+1}{2},n)\to (\frac{n-3}{2},n)\to (\frac{n+3}{2},n)\to \ldots \to (0,n)\to (n,n). \end{array}$$

**Fig 4:**
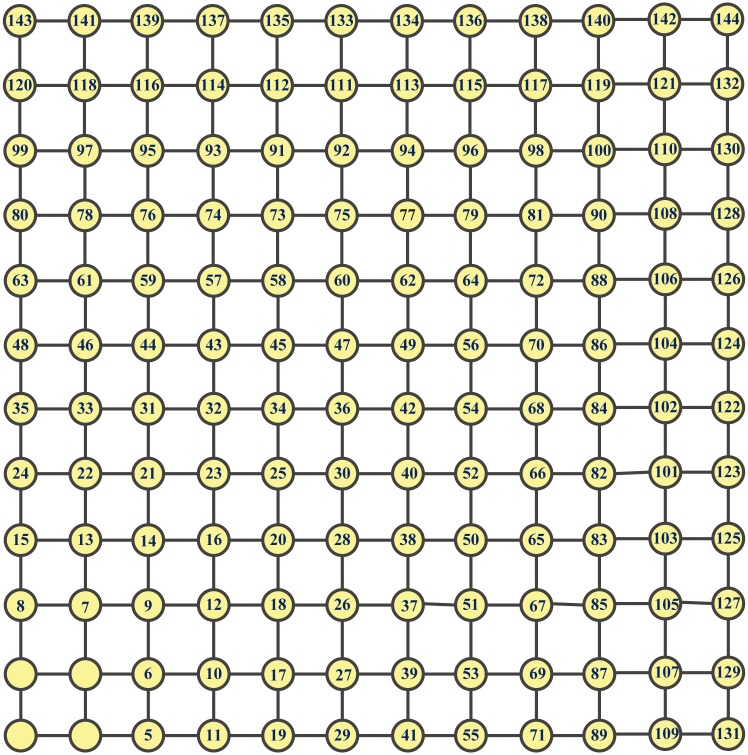
A proximity growth of GM_*PR*_(144).

**Fig 5:**
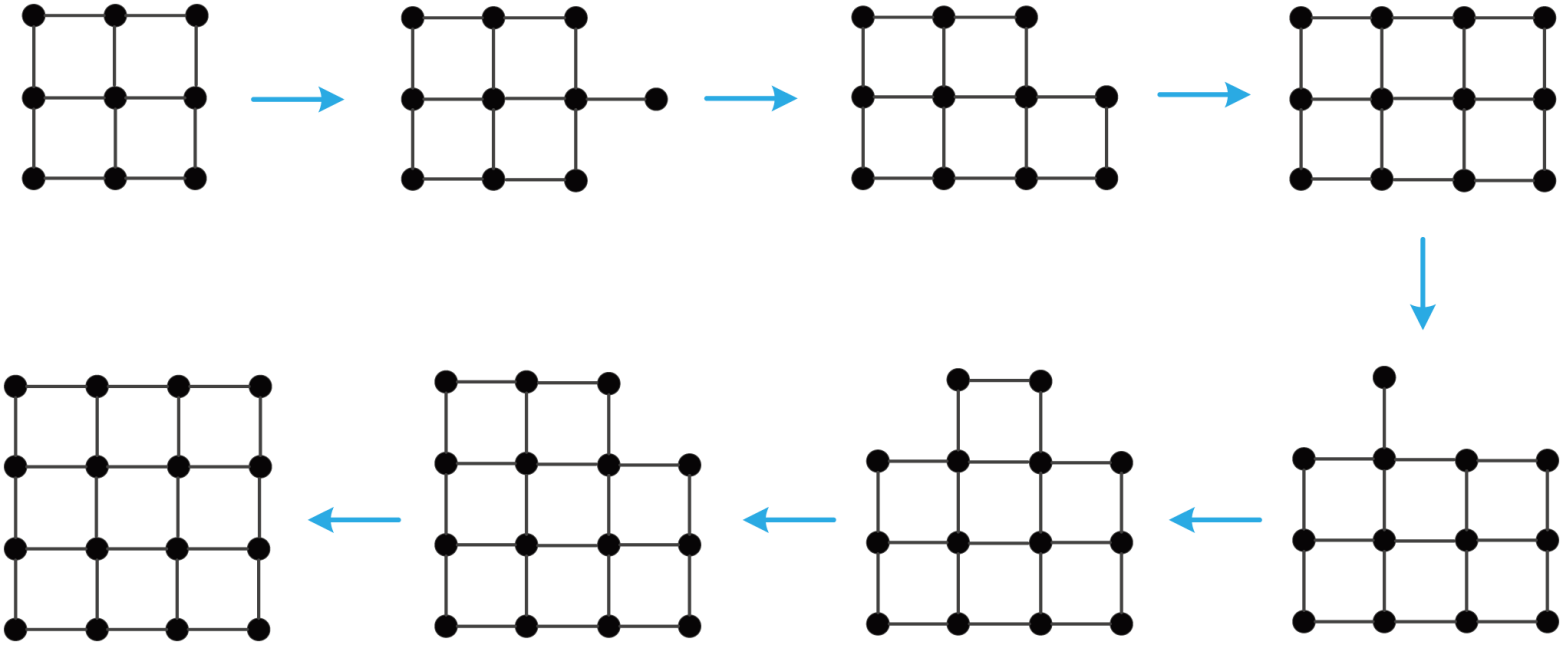
A proximity growth of M_3_ to M_4_.

Fig 4 shows a proximity growth of **G** **M**_*PR*_(144), where the numbers in the circles stand for the node-adding order. Fig 5 displays a proximity growth of **M**_3_ to **M**_4_. Intuitively, the PR-GMs are most robust. In the sequel, we shall compare the GMs grown up by optimizing some other measures of network robustness with the PR-GMs.

## 4 Algebraic connectivity-optimal growth of GMs

The *algebraic connectivity* (AC, for short) of a network *G*, denoted *α*(*G*), is defined as the second smallest Laplacian eigenvalues of *G*. The algebraic connectivity is widely recognized as a rational measure of network robustness [2–7, 11–13].

**Fig 6:**
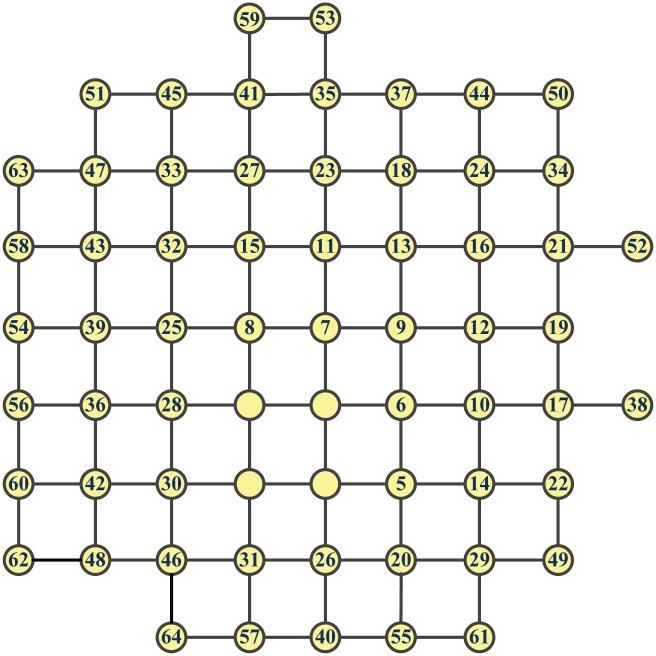
A stepwise AC-optimal growth of GM_*AC*_(64).

Let **GM**_*AC*_(*N*) denote an algebraic connectivity-optimal GM with *N* nodes. Numerical calculations give a AC-optimal growth of **GM**_*AC*_(64), see Fig 6. The following facts can be concluded from this figure.

**Fig 7:**
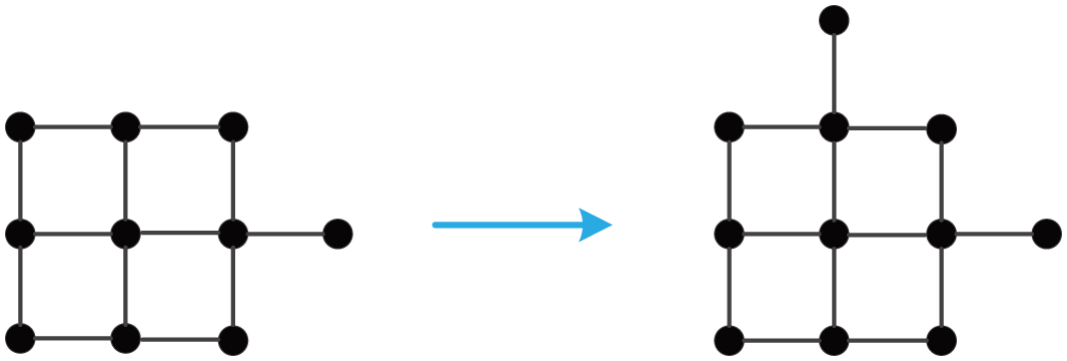
A AC-optimal growth of GM_*AC*_(10) to GM_*AC*_(11).

**Fig 8:**
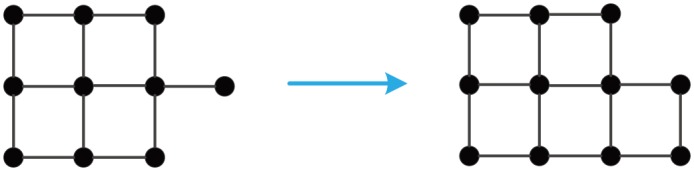
A proximity growth of GM_*PR*_(10) to GM_*PR*_(11).

**Fig 9:**
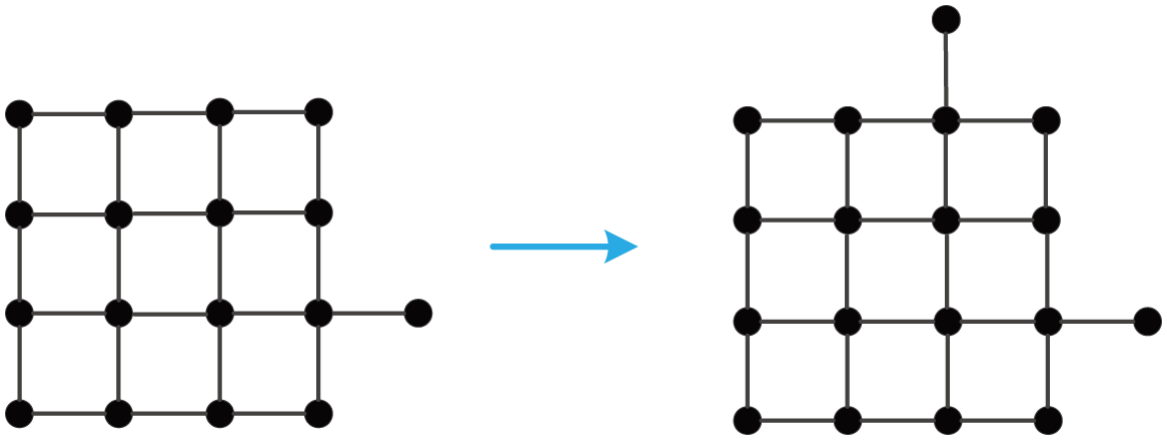
A AC-optimal growth of GM_*AC*_(17) to GM_*AC*_(18).

**Fig 10:**
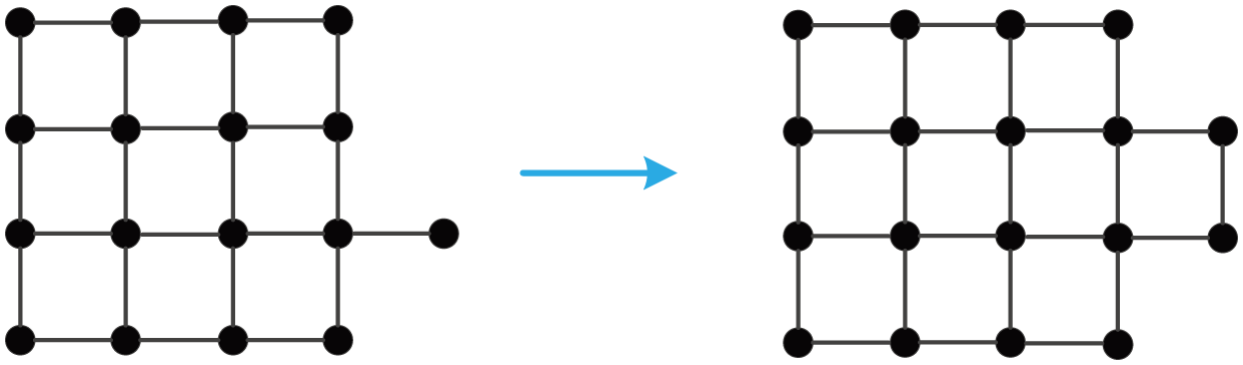
A proximity growth of GM_*PR*_(17) to GM_*PR*_(18).

1. For 4 ≤ *N* ≤ 10, **GM**_*AC*_(*N*) is isomorphic to **GM**_*PR*_(*N*).
2. **GM**_*AC*_(11) is not isomorphic to **GM**_*PR*_(11), see Figs 7 and 8. **GM**_*AC*_(11) is less robust than **GM**_*PR*_(11), because
  $$\kappa \left({\text{GM}}_{AC}\left(11\right)\right)=1<2=\kappa \left({\text{GM}}_{PR}\left(11\right)\right).$$
3. **GM**_*AC*_(18) is not isomorphic to **GM**_*PR*_(18), see Figs 9 and 10. **GM**_*AC*_(18) is less robust than **GM**_*PR*_(18), because
  $$\kappa \left({\text{GM}}_{AC}\left(18\right)\right)=1<2=\kappa \left({\text{GM}}_{PR}\left(18\right)\right).$$
4. Similar phenomena occur frequently in a AC-optimal growth of GMs.

The above discussions demonstrate that, at least in some situations, the algebraic connectivity is not suited to serve as a metric of network robustness. Hence, the utility of the algebraic connectivity as a metric of network tolerance is still in doubt.

## 5 Effective resistance-optimal growth of GMs

The effective resistance (ER, for short) of a network, denoted *ER*(*G*), is defined as follows. First, regard the network as an electrical network with one Ohm resistor on each link. Second, calculate the effective resistance between each pair of nodes by invoking the Kirchhoff’s circuit law. Third, sum up the effective resistances over all pairs of nodes to get the ER of the original network.

The effective resistance of a network has been advised as a measure of robustness of the network; the smaller the ER, the more robust the network [8]. The effective resistance outperforms the algebraic connectivity, because the former decreases strictly when a new edge is added to a network, whereas the latter may or may not rise up [30].

Let 0 = *λ*_1_ < *λ*_2_ ≤ … ≤ *λ*_*n*_ denote the Laplacian spectrum of a connected network *G*. Klein and Randić [30] found that the effective graph resistance of a connected network can be written as a function of all non-zero Laplacian eigenvalues of the network. Specifically,

$$ER\left(G\right)=n\sum_{k=2}^{n}\frac{1}{\lambda_{k}}$$

This equation offers a method for numerically calculating the ER of a network.

Let **G** **M**_*ER*_(*N*) denote an effective resistance-optimal GM with *N* nodes. calculations show that for 4 ≤ *N* ≤ 144, **G** **M**_*ER*_(*N*) is isomorphic to **G** **M**_*PR*_(*N*). Hence, it is concluded that the ER is a reasonable measure of network robustness.

## 6 Average edge betweenness-optimal growth of GMs

The *betweenness centrality* of an edge of a network is defined as the number of the node-pair shortest paths that go through the edge [31]. The notion of edge betweenness centrality was originally proposed by Girvan and Newman [32] to find the bottlenecks of a network; an edge with a high edge betweenness centrality score represents a bridge-like connector between two parts of a network, and the removal of which may affect the communication between many pairs of nodes [33, 34].

The *average edge betweenness* (AEB, for short) of a network *G*, denoted *AEB*(*G*) is defined as the arithmetic average of the betweenness centralities of all edges of *G* [8–10]. Ellens [9] suggest the AEB as a metric of network robustness, because, intuitively, the lower the AEB, the more robust the network.

Let $\bar{d}$(G) denote the average distance of a network *G*, then

$$AEB\left(G\right)=\frac{\nu \left(G\right)\left[\nu (G)-1\right]}{2\varepsilon (G)}\bar{d}\left(G\right),$$

where *ν*(*G*) and *ε*(*G*) denote the number of nodes and edges of *G*, respectively [9]. This equation offers a method for numerically calculating the AEB of a network.

Let **GM**_*AEB*_(*N*) denote an average edge betweenness-optimal GM with *N* nodes. calculations show that for 4 ≤ *N* ≤ 144, **GM**_*AEB*_(*N*) is isomorphic to **GM**_*PR*_(*N*). This partly justifies the AEB as a metric of network robustness.

## 7 Efficiency-optimal growth

The *efficiency* of a network *G* is defined as

$$EFF(G)=\frac{2}{\nu (G)\left[\nu (G)-1\right]}\sum_{\begin{array}{c} u,v\in V(G)\\ u\neq v \end{array}}\frac{1}{d_{G}(u,v)},$$

where *d*_*G*_(*u*, *v*) denotes the distance between nodes *u* and *v*. The notion of efficiency was originally proposed by Latora and Marchiori [35, 36] to characterize the closeness of a network. Ellens and Kooij [10] proposed to use the efficiency as a metric of network robustness, because, intuitively, the higher the efficiency, the more robust the network. One advantage of this measure is that it can be used for unconnected networks.

**Fig 11:**
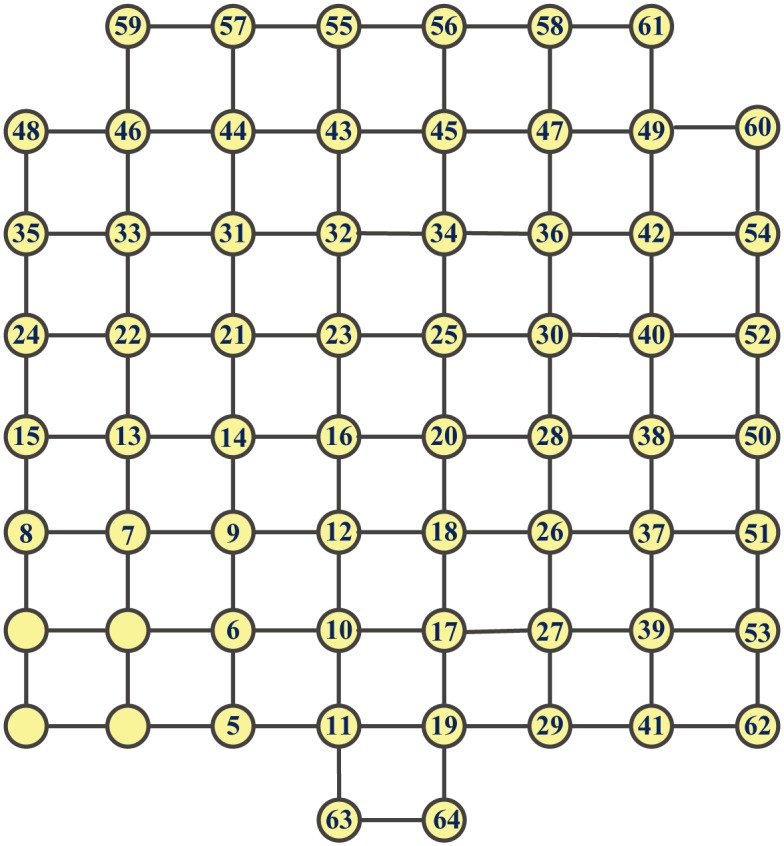
An efficiency-optimal growth of GM_*EFF*_(64).

Let **GM**_*EFF*_(*N*) denote an algebraic connectivity-optimal GM with *N* nodes. Numerical calculations give an efficiency-optimal growth of **G** **M**_*EFF*_(64), see Fig 11. The following facts can be derived from this figure.

**Fig 12:**
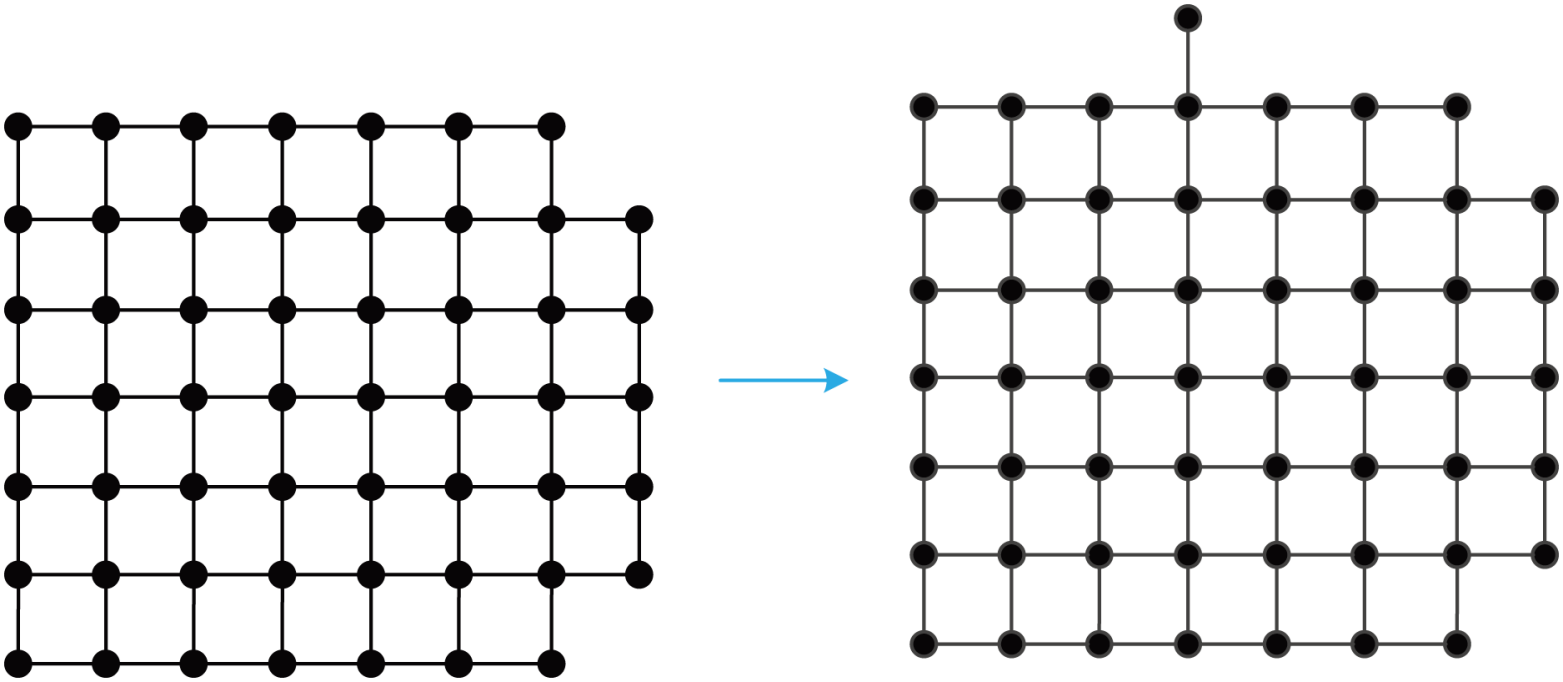
An efficiency-optimal growth of GM_*EFF*_(54) to GM_*EFF*_(55).

**Fig 13:**
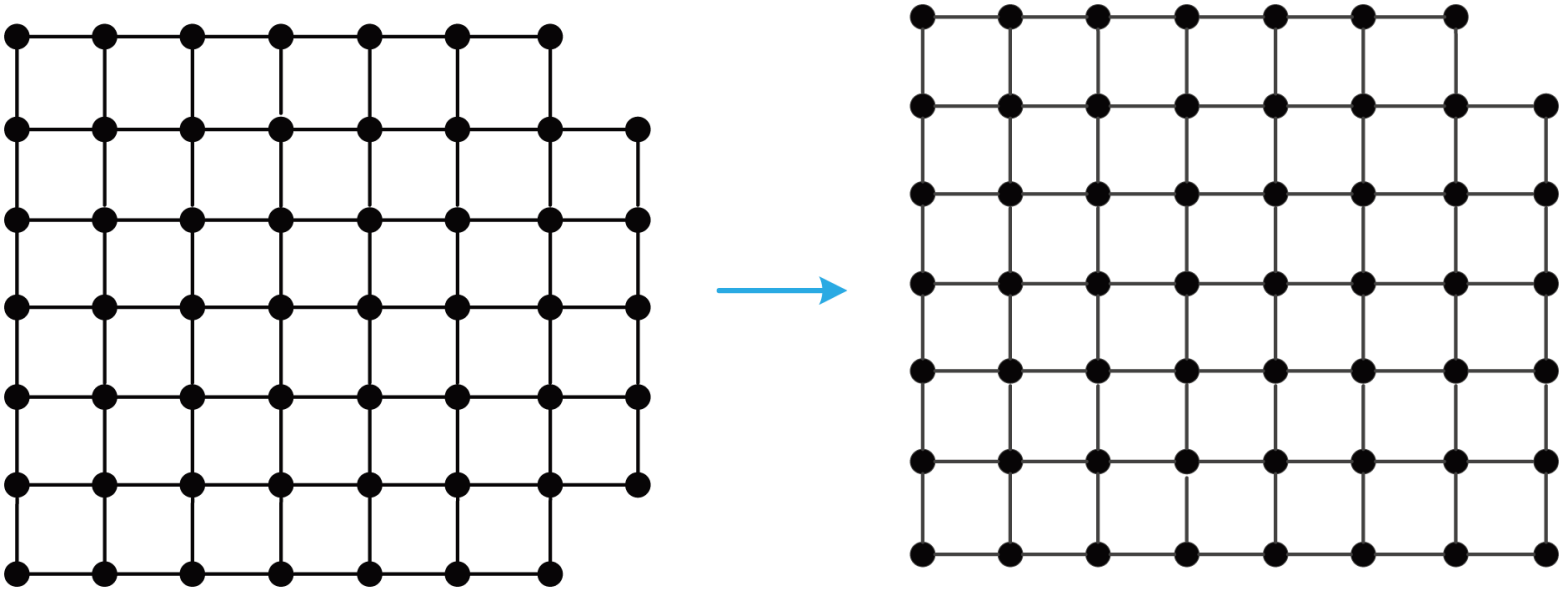
A proximity growth of GM_*PR*_(54) to GM_*PR*_(55).

1. Up to isomorphism, the efficiency-optimal growth of **GM**_*EFF*_(54) coincides with the proximity growth of **GM**_*PR*_(54).
2. **GM**_*EFF*_(55) is not isomorphic to **GM**_*PR*_(55), see Figs 12 and 13. **GM**_*EFF*_(55) is less robust than **GM**_*PR*_(55), because
  $$\kappa \left({\text{GM}}_{EFF}\left(55\right)\right)=1<2=\kappa \left({\text{GM}}_{PR}\left(55\right)\right).$$

The above discussions show that the utility of the efficiency as a metric of network robustness is limited.

## 8 Conclusions

This paper has addressed the rationality of four metrics of network robustness (the algebraic connectivity, the effective resistance, the average edge betweenness, and the efficiency) by investigating the robust growth of generalized meshes (GMs). A heuristic algorithm for the robust growth of GMs has been proposed. Some GMs have been grown up by optimizing a measure of network robustness. A comparative analysis shows that (1) the effective graph resistance and the average edge betweenness can serve as metrics of network robustness, (2) the utility of the efficiency as a metric of network robustness is limited, and (3) the utility of the algebraic connectivity as a metric of network robustness is highly in doubt.

In our opinion, this work should be extended to other types of networks, such as the hexagonal networks [37–39] and the honeycomb networks [40–43].
